# Clinical courses and complications of young adults with Autosomal Recessive Polycystic Kidney Disease (ARPKD)

**DOI:** 10.1038/s41598-019-43488-w

**Published:** 2019-05-28

**Authors:** Kathrin Burgmaier, Samuel Kilian, Bert Bammens, Thomas Benzing, Heiko Billing, Anja Büscher, Matthias Galiano, Franziska Grundmann, Günter Klaus, Djalila Mekahli, Laurence Michel-Calemard, Gordana Milosevski-Lomic, Bruno Ranchin, Katja Sauerstein, Susanne Schaefer, Rukshana Shroff, Rosalie Sterenborg, Sarah Verbeeck, Lutz T. Weber, Dorota Wicher, Elke Wühl, Jörg Dötsch, Franz Schaefer, Max C. Liebau

**Affiliations:** 1 Department of Pediatrics, University Hospital of Cologne, Cologne, Germany; 2 Institute of Medical Biometry and Informatics, University of Heidelberg, Heidelberg, Germany; 3 Department of Microbiology and Immunology, Laboratory of Nephrology, KU Leuven, Leuven, Belgium; 4 Department of Nephrology, Dialysis and Renal Transplantation, University Hospitals Leuven, Leuven, Belgium; 5 Department II of Internal Medicine and Center for Molecular Medicine Cologne, University of Cologne, Cologne, Germany; 6 Cologne Excellence Cluster on Cellular Stress Responses in Aging Associated Diseases, University of Cologne, Cologne, Germany; 7 Children’s University Hospital Tuebingen, Department of General Pediatrics and Hematology/Oncology, Tuebingen, Germany; 8 Department of Pediatrics II, University Hospital Essen, Essen, Germany; 9 Department of Pediatrics and Adolescent Medicine, Hospital of the Friedrich-Alexander-University Erlangen-Nürnberg (FAU), Erlangen, Germany; 10 KfH Center of Paediatric Nephrology, University Hospital of Marburg, Marburg, Germany; 11 PKD Research Group, Department of Development and Regeneration, KU Leuven, Leuven, Belgium; 12 Department of Pediatric Nephrology, University Hospitals Leuven, Leuven, Belgium; 13 Service Biochimie Biologie Moléculaire Grand Est, Hospices Civils de Lyon, Bron Cedex, France; 14 Department of Nephrology, University Children’s Hospital, Belgrade, Serbia; 15 Pediatric Nephrology Unit, Hôpital Femme Mere Enfant, Hospices Civils de Lyon, Lyon, France; 16 Division of Pediatric Nephrology, Center for Pediatrics and Adolescent Medicine, University of Heidelberg, Heidelberg, Germany; 17 Great Ormond Street Hospital for Children NHS Foundation Trust, London, United Kingdom; 18 Department of Medical Genetics, The Children’s Memorial Health Institute, Warsaw, Poland; 19 Center for Molecular Medicine Cologne, University of Cologne, Cologne, Germany

**Keywords:** Polycystic kidney disease, Liver fibrosis

## Abstract

Autosomal recessive polycystic kidney disease (ARPKD) is a severe pediatric hepatorenal disorder with pronounced phenotypic variability. A substantial number of patients with early diagnosis reaches adulthood and some patients are not diagnosed until adulthood. Yet, clinical knowledge about adult ARPKD patients is scarce. Here, we describe forty-nine patients with longitudinal follow-up into young adulthood that were identified in the international ARPKD cohort study ARegPKD. Forty-five patients were evaluated in a cross-sectional analysis at a mean age of 21.4 (±3.3) years describing hepatorenal findings. Renal function of native kidneys was within CKD stages 1 to 3 in more than 50% of the patients. Symptoms of hepatic involvement were frequently detected. Fourteen (31%) patients had undergone kidney transplantation and six patients (13%) had undergone liver transplantation or combined liver and kidney transplantation prior to the visit revealing a wide variability of clinical courses. Hepatorenal involvement and preceding complications in other organs were also evaluated in a time-to-event analysis. In summary, we characterize the broad clinical spectrum of young adult ARPKD patients. Importantly, many patients have a stable renal and hepatic situation in young adulthood. ARPKD should also be considered as a differential diagnosis in young adults with fibrocystic hepatorenal disease.

## Introduction

Autosomal recessive polycystic kidney disease (ARPKD) is a rare form of polycystic kidney disease, a hepatorenal disorder most frequently presenting during childhood. The disease is caused by variants in the *Polycystic Kidney and Hepatic Disease 1* (*PKHD1*) gene or, rarely, in the *DAZ interacting protein 1-like* gene (*DZIP1L*)^1–4^. The classic ARPKD phenotype is characterized by early-onset disease with bilateral enlargement of the kidneys and impairment of renal function as well as congenital hepatic fibrosis with subsequent portal hypertension^5^. ARPKD represents one of the leading causes of pediatric dialysis-dependency and pediatric kidney-, liver- or combined liver and kidney transplantation (KTx, LTx, CLKTx)^6^. Yet, there is major inter- and even intra-familiar clinical variability^7^. The correlation between hepatic and renal symptoms currently remains unclear^7,8^. While renal involvement typically presents early in life or even prenatally, liver involvement tends to manifest later, typically with progressive hepatic fibrosis and portal hypertension^9,10^.

In rare cases, both renal and hepatic involvement may present in late adolescence or even in adulthood. Furthermore, thanks to medical progress in neonatology, pediatric nephrology, pediatric hepatology and pediatric transplant surgery, a substantial number of ARPKD patients reaches adulthood. The diagnosis and treatment of adult ARPKD patients can be particularly challenging due to the low prevalence of the disease in general, partly non-typical disease manifestations^11,12^ and a very low incidence of initial diagnoses of ARPKD in adulthood. In the literature, we can only refer to small case series or single case reports on adult ARPKD patients^12–20^.

Here, we describe the real-world clinical spectrum of a large adult ARPKD cohort based on the international ARPKD registry and cohort study ARegPKD^21,22^.

## Results

### Patient characteristics

From July 2013 to February 2018, 470 patients with the clinical diagnosis of ARPKD that were treated at 63 centres in 19 different countries were included in the ARegPKD registry study (135 patients from Germany, 98 from Turkey, 80 from Poland, 27 from UK, 130 from other countries). Eighteen patients were excluded due to failure to comply with the inclusion/exclusion criteria or insufficient data quality. Of the remaining 452 patients, 49 patients (11%) with documented visits at age ≥ 18 years were available for data analysis. These 49 patients derived from 24 centres in 10 countries (23 patients from Germany, 9 from Belgium, 3 each from Poland, Turkey, France and the United Kingdom, 5 from other countries). For cross-sectional analysis and time-to-event-analysis of complications or organ manifestations, data sets of 45 adult patients with at least one visit at age 18 to 34 years were available. For four patients there were no suitable documented visits in this defined age frame. The number of informative cases for specific aspects of the cross-sectional analysis varied according to the availability of clinical information as provided by the participating centres. A flow chart of the patient selection process is depicted in Supplementary Fig. S1.

General anthropometric patients’ characteristics at analysed visit are shown in Table 1. Due to the definition of analysis time point, patients were young adults with a median age of 19.9 years (range 18–29) and a mean age of 21.4 (±3.3) years.

**Table 1:** Patient characteristics at time of cross-sectional analysis.

	45 adult patients with closest visit to age of 26 years (n/total n informative cases (%) OR mean (±SD) of informative cases)
**General information**	
Age at visit (years)	21.4 (±3.3)
Sex (male)	24/45 (53.3%)
Height (cm; n = 29)	170.9 (±10.2)
Weight (kg; n = 35)	65.9 (±15.4)
BMI (kg/m²; n = 29)	21.6 (±3.0)

### Renal phenotype

**Figure 1:**
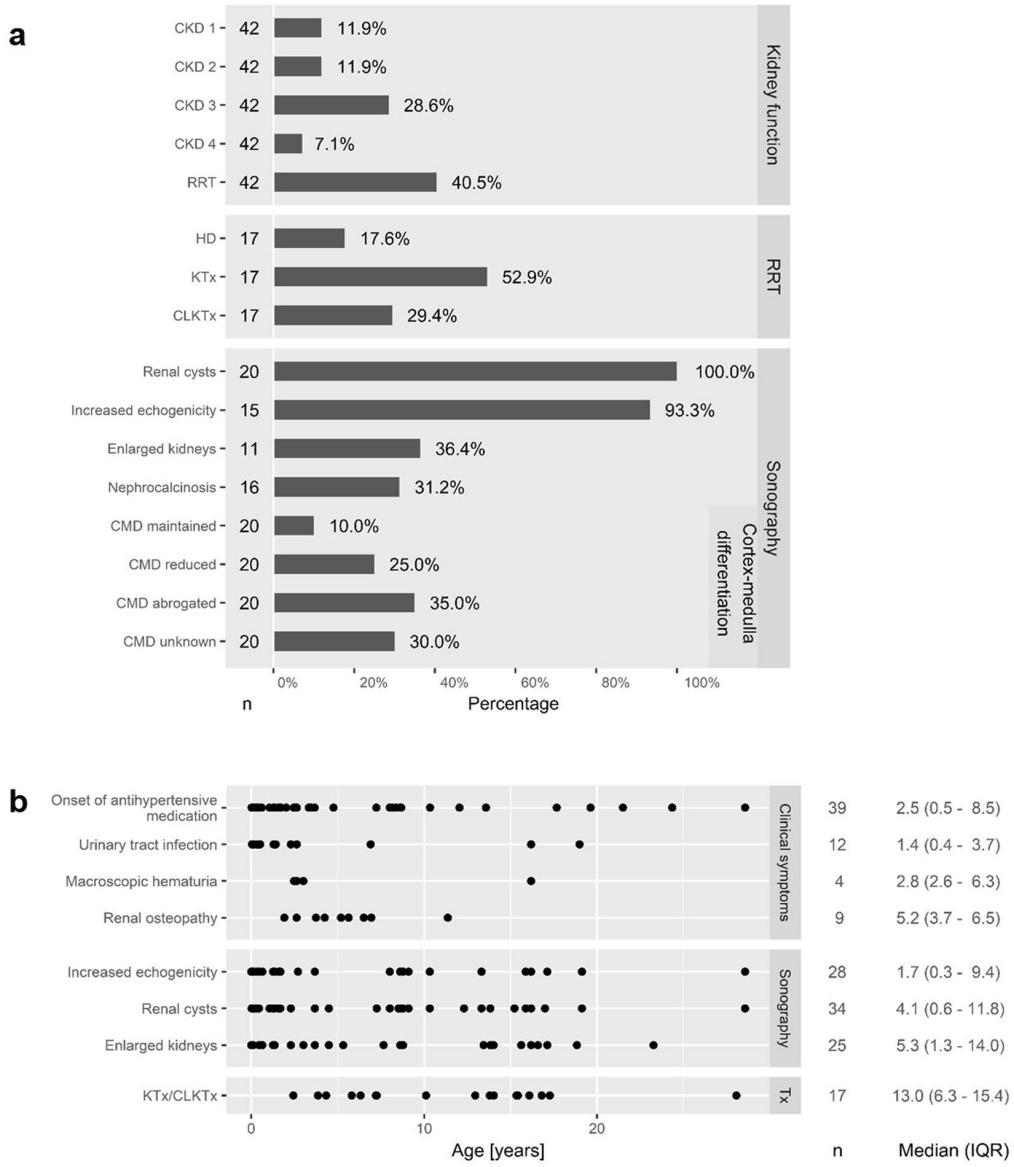
(**a**) Renal phenotype at cross-sectional analysis of 45 adult patients regarding kidney function and sonographic abnormalities. HD = hemodialysis, KTx = kidney transplant graft survival, CLKTx = combined liver and kidney transplant graft survival, CMD = cortex-medulla differentiation, n = number of informative cases with sufficient available data for the specific analysis. (**b**) Dot plot of age at first documentation of renal abnormalities regarding clinical and sonographic characteristics with median and IQR. Events after KTx/CLKTx were excluded; n = number of informative cases.

More than half of the patients with available laboratory values at the analysed visit (22/42, 52%) showed native kidney function within chronic kidney disease (CKD) stages 1 to 3 (Fig. 1a). Seventeen out of 42 (41%) patients received renal replacement therapy (RRT) prior to the analysed visit with a median age at onset of 11.0 years (IQR 5.3–15.3). Seventeen patients underwent at least one KTx or CLKTx. Median age at first KTx/CLKTx (n = 17) was 13.0 years (IQR 6.3–15.4; Fig. 1b). Two-year kidney graft survival was 88% for KTx and 80% for CLKTx.

Mean office blood pressure (n = 38) at the analysed visit was 125 (±16) mmHg to 73 (±13) mmHg for the overall cohort. All but 7 patients (38/45, 84%) required at least one antihypertensive substance with 6/45 (13%) taking agents from three or four different substance classes. The most frequently applied substances were ACE-inhibitors (25/45, 56%) and beta blockers (15/45, 33%).

Mean pole-to-pole (PTP) lengths of native kidneys (n = 10) were 11.5 cm (±2.7, range 7.1–15.0) for the right kidney and 11.6 cm (±3.3, range 7.1–16.0) for the left kidney as determined by ultrasound. Mean PTP lengths in patients without RRT (n = 7) were 12.6 (±2.0) cm for the right and 13.2 (±2.5) cm for the left kidney. Patients with previous RRT (n = 3) showed smaller kidneys with a mean PTP length of 8.8 (±2.4) cm for the right and 7.7 (±0.8) cm for the left kidney. Data on ultrasound-determined kidney volume were scarce and varied grossly with mean volumes of 327.0 ml (±121.8, range 205.0–441.0 ml, n = 4) for the right and 292.0 ml (±102.2, range 230.0–410.0 ml, n = 3) for the left native kidney. Sonographic morphology of the native kidneys encompassed renal cysts, increased echogenicity and reduced or abrogated cortex-medulla differentiation (Fig. 1a). All patients with evaluable ultrasound analyses showed detectable renal cysts with most patients showing multiple bilateral cysts (>10 cysts per kidney). The remaining patients were indicated to show few separated cysts per kidney (up to 3). One patient did not have detectable cysts in the right kidney, but two cysts in the left kidney despite being compound heterozygous for two *PKHD1* missense variants.

Time-to-event analysis of renal disease manifestations revealed urinary tract infections, macroscopic hematuria and even renal osteopathy early in childhood, while documentation of arterial hypertension clustered slightly wider over childhood into early adolescence. With regards to sonographic abnormalities, increased echogenicity and renal cysts were usually reported prior to kidney enlargement (Fig. 1b).

### Liver phenotype

**Figure 2:**
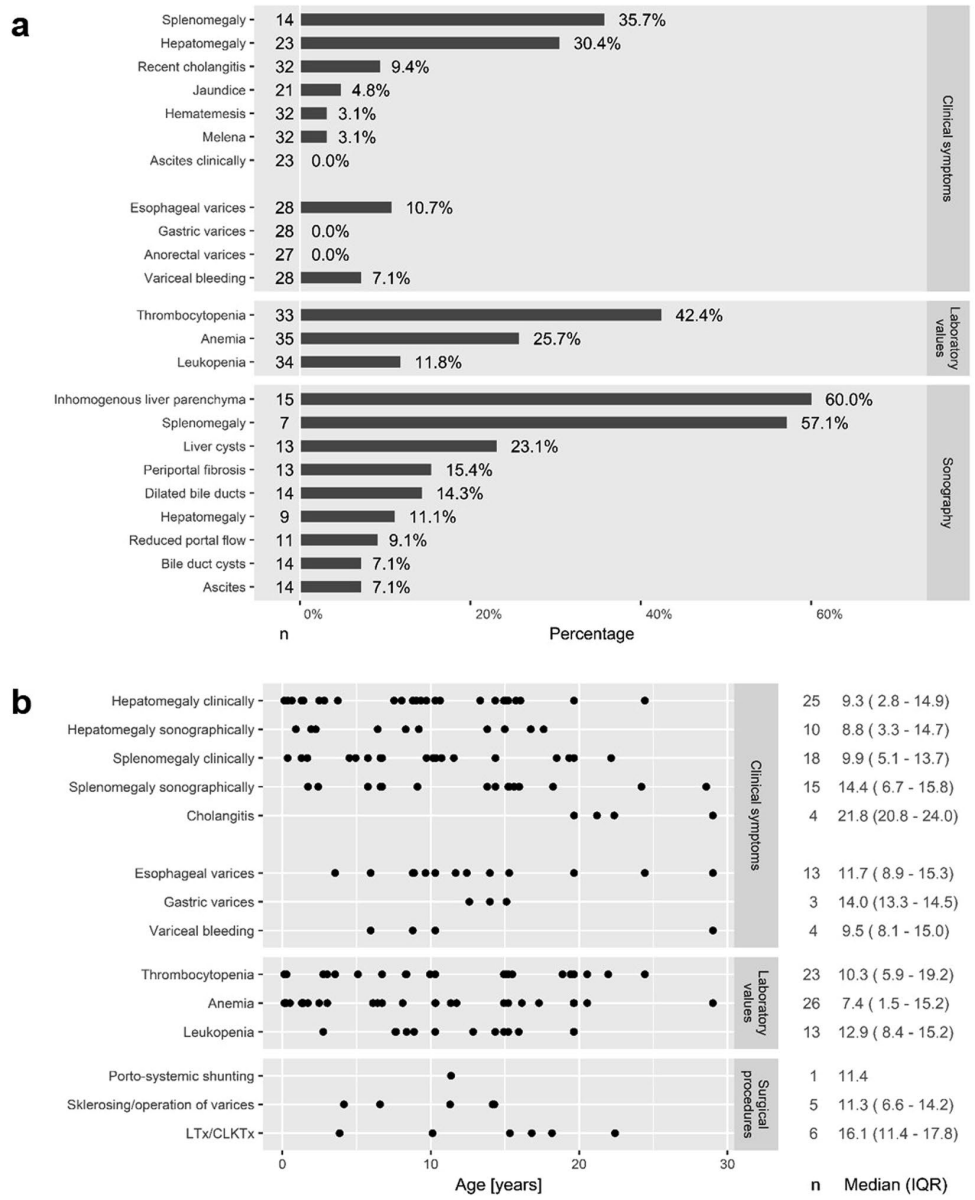
(**a**) Liver-related clinical symptoms and sonographic abnormalities of 39 adult patients without previous LTx/CLKTx at time of cross-sectional analysis; n = number of informative cases. (**b**) Dot plot of age at first documentation of signs of portal hypertension and/or liver complications with median and IQR. Events after LTx/CLKTx were excluded; n = number of informative cases.

The liver phenotype of the 39 patients without previous transplantation at the analysed visit was evaluated according to reported past medical history (n = 32), physical examination (n = 23), laboratory findings (n = 35), and sonographic results (n = 15; Fig. 2a). Six patients underwent LTx or CLKTx prior to the analysed visit. Median age at LTx/CLKTx (n = 6) was 16.1 years (IQR 11.4–17.8). In almost one third of patients hepatomegaly and splenomegaly were detected by physical examination. Esophageal varices (3/28) and recent diagnosis of cholangitis since the last documented visit (3/32) were reported in some cases. There were no reports of gastric or anorectal varices at the analysed visit. Sonographic abnormalities were more frequently detected with 9/15 (60%) patients showing inhomogeneous liver parenchyma and 2/14 (14%) patients with reports of dilated bile ducts. Liver cysts were described in 3/13 (23%) patients (Fig. 2a). Mean native liver size in the medioclavicular line (MCL; n = 9) was 13.7 (±3.4) cm, i.e. within the normal range. One out of 9 patients showed hepatomegaly on ultrasound. Mean sonographic spleen size (n = 7, 14.8 ± 2.9 cm) was above the upper normal limit of 13 cm in more than half of the patients without previous LTx/CLKTx. Thrombocytopenia (platelets < 150.000/µl) occurred in 42% of these patients. The means (SD) of liver enzymes in patients without previous LTx/CLKTx were within normal ranges with only few patients showing mildly elevated values (less than two times upper limit of normal): alanine-aminotransferase (ALT; upper limit of normal (ULN) 49 U/l in males, 34 U/l in females) was elevated in 3/28 (11%) patients, aspartate-aminotransferase (AST; ULN 49 U/l in males, 34 U/l in females) in 1/26 (4%) patients and gamma-glutamyltransferase (GGT; ULN 65 U/l in males, 38 U/l in females) in 9/24 (38%) patients. Values for total bilirubin (ULN 1.0 mg/dl) were elevated in 2/17 (12%) patients. Evaluation of synthetic liver function revealed normal values for cholinesterase (n = 3; lower limit of normal (LLN) 4.7 kU/l in males, 4.0 kU/l in females) and serum albumin (n = 23; LLN 35 g/l) in all examined patients and only a single patient with mildly increased International Normalized Ratio (INR) (out of 10 patients with available data; ULN 1.25; maximum INR 1.8).

Time to first signs of hepatic involvement (hepatomegaly, cholangitis) and portal hypertension (splenomegaly, thrombocytopenia, anemia with hemoglobin <11.0 g/dl, leukopenia with white blood cell count <3500/µl, varices or variceal bleeding, sclerosing/operation of varices, implementation of portosystemic shunts) is indicated as dot plot in Fig. 2b. The median age at first documentation of thrombocytopenia was lower than the median age at first documentation of collateral blood flow and cholangitis. Cholangitis occurred in only four patients of this specific cohort at a median age of 21.8 years (IQR 20.8–24).

### Primary manifestations and establishment of diagnosis

One quarter of patients (7/28) showed prenatal anomalies with detection of oligo- or anhydramnios (5/27, 19%), renal cysts (2/25, 8%) and increased renal echogenicity (1/24, 4%). There was no documentation of prenatal hepatic anomalies. Thirteen of 32 (41%) patients showed some form of perinatal problems with 6/28 (21%) requiring assisted breathing or ventilation.

**Figure 3:**
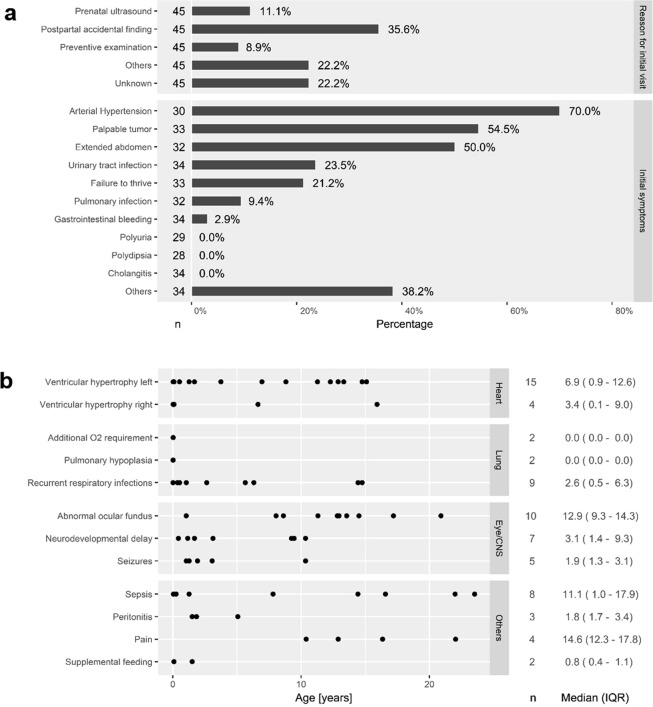
(**a**) Reasons for initial visit of 45 adult patients and initial symptoms at initial presentation (multiple answering possible). (**b**) Dot plot of age at first documentation of specific extrarenal and extrahepatic organ manifestations or complications with median and IQR; n = number of informative cases.

Approximately one third (16/45) of patients were primarily diagnosed by postpartal incidental findings (Fig. 3a). Other reasons for initial visits at a doctor included diagnostic workup for failure to thrive, cardiac failure, macroscopic hematuria, urinary tract infection or respiratory distress syndrome. Median age at diagnosis (n = 37) was 0.5 (IQR 0.1–1.5) years with a range of −0.3 to 25 years indicating that one quarter of all patients received their diagnosis pre- or perinatally.

Initial clinical symptoms most frequently encompassed arterial hypertension, palpable kidneys/tumor and extended abdomen. Almost one quarter of patients (8/34, 24%) initially suffered from urinary tract infections. Only one patient (1/34, 3%) primarily presented with gastrointestinal bleeding. Other symptoms encompassed acute renal failure, edema, macroscopic hematuria, enuresis/pollakisuria, icterus/dystrophy, vomiting, intussusception, inguinal hernia, pneumothorax and recurrent infections (Fig. 3a).

*PKHD1* sequencing was performed in 20 of the 45 patients included in the cross-sectional analysis with variant detection in 18/20 (90%) patients and a variant detection rate of 32/40 alleles (80%), in keeping with other studies^7,11^. Of the 18 patients with *PKHD1* variants, the diagnosis of ARPKD was confirmed in 7/18 patients (39%), remains probable in 4/18 (22%) and unknown in 7/18 patients (39%) according to the criteria of the American College of Medical Genetics^23^.

### Other complications

The time to first documentation of extrarenal and extrahepatic complications is indicated in Fig. 3b. First documentation of left ventricular hypertrophy, recurrent respiratory infections and neurological abnormalities clustered mainly within the first decade of life. First documentation of abnormal ocular fundus (hypertensive retinopathy, papilledema), abdominal pain and septic episodes showed a wider distribution. Remarkably, no reports of cerebral aneurysms or cholangiocarcinoma have been documented in our cohort so far.

### Organ replacement therapy

**Figure 4:**
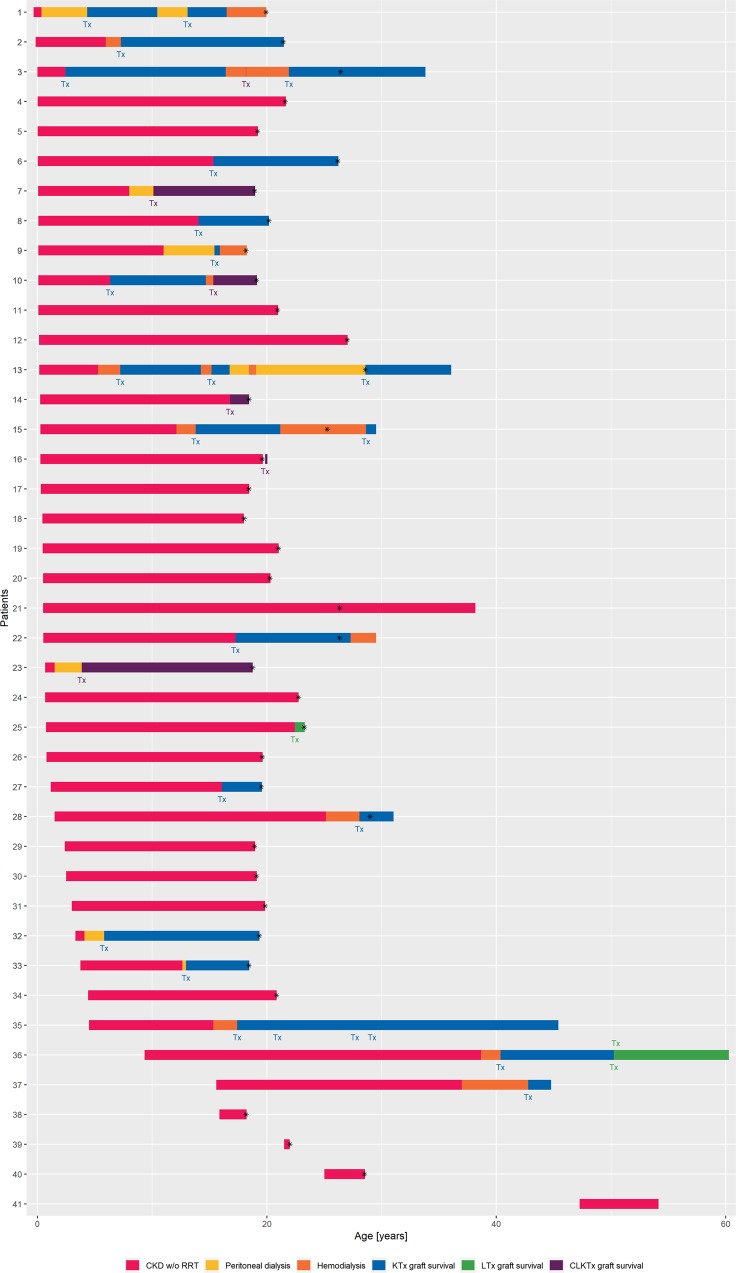
Individual courses of CKD and organ replacement therapies of 49 patients with available longitudinal data. Bars indicate length of CKD, dialysis or transplant graft survival. Bars end with age of last documented visit. Asterix indicates analysed visit for cross-sectional analysis. None of the reported patients deceased. “Tx” indicates timepoint of transplantation in color coding. Note that four patients (No. 35, 36, 37, 41) did not have suitable documented visits for cross-sectional analysis.

Eighteen out of 45 patients (40%) underwent at least one, in total 24 organ transplantations (Tx) prior to the timepoint of cross-sectional analysis with 14/45 patients (31%) receiving 18 kidney grafts, 1/45 patient (2%) undergoing liver Tx and 5/45 patients (11%) undergoing combined liver and kidney Tx (CLKTx). Two of the patients received an isolated KTx first and required CLKTx afterwards. Five out of 24 organ donations (21%) were living related donor organ donations, 19/24 (79%) were deceased donor organ donations. No patient remained on dialysis, all patients with requirement of RRT underwent renal transplantation during the course of disease. Median age at first KTx (n = 14) was 13.4 (range 2.4–28) years, at first CLKTx (n = 5) 15.3 (range 3.9–18.2) years and the one LTx was performed at age of 22.4 years. Median age at onset of RRT (HD, PD, KTx, CLKTx; n = 17) was 11 (range 0.4–25) years. Indications for LTx or CLKTx (n = 6) were severe variceal bleeding episodes (n = 2) as well as recurrent cholangitis and sepsis, hypersplenism and cholestasis in one case each. As pointed out above graft survival was within expected ranges. Individual long-term clinical courses of patients are presented in Fig. 4.

## Discussion

ARPKD typically is a disease of early childhood. Yet, given the progress in neonatal and pediatric care more and more patients reach adulthood. The clinical courses and complications in adulthood have not been described in detail. We may thus be facing novel challenges and even newly detected symptoms for this well-known disease as more and more adult ARPKD patients are being treated. We therefore performed a cross-sectional analysis with additional longitudinal data on young adult patients.

Our cohort of 45 young adults included in the cross-sectional analysis showed body dimensions within normal range despite the long-term disease courses. Importantly, almost half of the cohort showed stable native renal function in CKD stages 1–3. While some degree of survivor bias cannot be excluded, these findings are in accordance with previous observations^7^ and suggest that kidney function in ARPKD may be better preserved than widely anticipated. The additional renal disease manifestations documented in our cohort also are consistent with previous descriptions^7,11^. Hypertension was very common and multi-drug antihypertensive therapy was required in some patients.

Interestingly, kidney length at young adult age varied grossly, emphasizing the major variability of kidney size in ARPKD patients^15^. Notably, reported kidney lengths were only moderately above mean normal values also in those patients without prior renal replacement therapy. Our data may be subject to a detection bias since documentation of both height and exact pole-to-pole length was required which was only available for a small subgroup. Still the documented data suggest that clinicians may not necessarily expect increased kidney lengths in young adult ARPKD patients.

Liver disease appeared to present later in life. Hepatic disease in ARPKD comes with two main presentations. On the one hand portal hypertension may develop as a consequence of progressive hepatic fibrosis. Splenomegaly was documented only in one third of patients based on clinical examination but in 57% according to ultrasound. Since 42% of this sub-cohort of patients without previous LTx showed thrombocytopenia as a marker of hypersplenism, sonographic detection of splenomegaly may be more sensitive and appropriate. In contrast to this, hepatomegaly was clinically diagnosed in 30% of the patients, but sonographically confirmed only in 11% of all patients without previous LTx. A reason might be that the clinical detection of the liver margin below the right costal arch can be misinterpreted as hepatomegaly in the setting of enlarged kidneys.

A second aspect of liver involvement in ARPKD is cholangitis. Almost 10% of reported patients suffered from recent cholangitis at the timepoint of the cross-sectional study. The late presentation of cholangitis in our cohort may appear to be a sign of weak hepatic involvement in the group analysed here. Nonetheless, six of the 45 patients received CLKTx or LTx during childhood and adolescence.

Serum liver function tests were only slightly elevated in a minor subcohort of patients. Standard liver function tests may not adequately reflect liver involvement, a finding in line with previous data^11^.

A potential correlation between renal and hepatic phenotypes in ARPKD has been discussed^7^. In our cohort, there are two adult patients with a predominant liver phenotype: one patient (No. 25, Fig. 4) required isolated LTx at the age of 22.4 years while kidney function was still well preserved (CKD stage 2). Another patient (No. 41, Fig. 4) suffered from gastrointestinal bleeding at the age of 36 years with mild morphological renal changes despite the detection of three *PKHD1* variants including a truncating variant. In the rest of the cohort, we observed a tendency towards concomitant renal and hepatic phenotype with a greater proportion of hepatic manifestation in those patients who also required RRT.

Regarding extrarenal and extrahepatic organ manifestation we observed different patterns: while severely affected toddlers presented with pulmonary complications including right ventricular hypertrophy, requirement of supplemental feeding and neurodevelopmental delay in the first three years of life, reports of arterial hypertension and its complications span the first two decades. Episodes of sepsis span broadly over three decades. Interestingly, in our young adult cohort we did not have reports on cholangiocarcinoma or cerebral aneurysms. One possible influencing factor might be the young age of the majority of reported patients.

Three quarters of the cohort received their diagnosis within the first two years of life. The case of patient No. 41 (Fig. 4) stands out from the rest with genetic confirmation of ARPKD diagnosis at the age of 54 years showing a single cyst in one kidney with otherwise normal renal sonographic findings. In case of late manifesting disease, diagnosis of ARPKD can be challenging since the sonographic pattern can present atypically. Genetic testing may be required to establish the correct diagnosis.

Our study faces a number of limitations: This is a purely descriptive study on a very specialized subgroup of patients suffering from a rare disease. We also expect a selection bias as ARegPKD encompasses considerably more pediatric than adult centres. In addition, the ARegPKD consortium comprises mainly tertiary care centres and patients with milder phenotype not requiring renal or liver replacement therapy might be preferentially treated at smaller centres not participating in our registry. Furthermore, in less severely affected adult ARPKD patients this diagnosis may sometimes not be considered due to non-typical presentation, and severely affected deceased patients might not be included retrospectively. Also, genetic confirmation of the clinical diagnosis was performed in less than half of the population. Finally, datasets of this observational study were based on the documentation of daily clinical work and the voluntary entry of data by the participating centres and varied in clinical deepness resulting in variable numbers of informative cases for specific analyses. The voluntary reporting may introduce selection bias regarding reporting centres as well as reported patients. The data thus represent a “real world” clinical setting. For some aspects the numbers thus remained small and should be considered as a first step to gain insights in the broad clinical picture.

The limitations are in part compensated by the relatively large number of young adult ARPKD patients, a very specialized subcohort of a rare disease, with description of the phenotypic spectrum. Hence, our study provides important information for attending physicians in a multi-disciplinary setting for both early-manifesting adult ARPKD patients with multiple medical issues as well as late-manifesting patients with atypical presentation or findings. An important conclusion from our data is that ARPKD should also be considered as a differential diagnosis in adult patients with cystic kidney disease with or without renal replacement therapy. Signs of hepatic involvement and portal hypertension such as thrombocytopenia or splenomegaly may be helpful for clinical guidance. A high level of suspicion is required to identify adult ARPKD patients and a specific work-up of liver involvement including the search for signs of portal hypertension should be considered in adult patients with cystic kidney disease.

## Methods

### Registry

Pediatric and adult patients with the clinical diagnosis of ARPKD were enrolled and followed in the international ARegPKD cohort study according to the clinical criteria of the protocol previously described^21,22^. The study protocol was approved by the Ethics Committee of the Faculty of Medicine of Cologne University and the Institutional Review Boards of participating sites. Approving ethics committees/institutional review boards of the participating co-authors of this subproject were: Ethics Committee of the Heidelberg University Clinic, Heidelberg, Germany; Commissie Medische Ethiek UZ KU Leuven/Onderzoek, Leuven, Belgium; Ethics Committee at the Medical Faculty and at the University Hospital of Tuebingen, Tuebingen, Germany; Ethics Committee of the Faculty of Medicine of the University Duisburg-Essen, Essen, Germany; Ethics Committee of the Friedrich-Alexander-University Erlangen-Nuremberg, Erlangen, Germany; Ethics Committee, The Phillips University Marburg, Marburg, Germany; Comité d’Éthique du CHU de Lyon, Lyon, France; Ethic committee of University Children’s Hospital, Belgrade, Serbia; Bloomsbury Research Ethics Committee, London, United Kingdom; Children’s Memorial Health Institute Bioethical Committee, Warsaw, Poland. Subject pseudonymisation is performed at the local centre after written informed consent from the participants and/or their legal guardians in accordance with local regulation. In brief, a basic dataset and follow-up visits are documented in ARegPKD. Pro- and retrospective visits are scheduled to be entered annually, but documentation at flexible time intervals is possible. While disease-defining questions require obligatory entry, data entry of items on clinical information is flexible and numbers therefore vary. Details on the collected data and data quality control were depicted previously^21,22^.

For genetic analysis all reported *PKHD1* variants were classified with regard to pathogenicity according to the revised criteria of the American College of Medical Genetics^23^. Estimated glomerular filtration rates (eGFR) were calculated according to full age spectrum (FAS) formula^24^. Results of laboratory testing were considered if performed not more than 12 months prior to visit date, results of sonographic examinations if performed not more than 24 months prior to visit date and if examinations were performed at age ≥ 18.0 years. Sonographic kidney enlargement was defined as bilateral pole-to-pole-length (PTP) > mean + 2SD for pediatric and adult measurements according to normal values for children^25,26^ and adults^27^. Sonography-based kidney volumes were calculated according to the ellipsoid formula (length × width × depth × π/6). Liver size in medioclavicular line (MCL) was interpreted according to available pediatric and adult cohorts^28,29^ with MCL > mean + 2SD (for children) and MCL > 17.4 cm (for adults) indicating hepatomegaly^29^. Sonographic splenomegaly was diagnosed according to the upper limits suggested by pediatric and adult cohort studies^30–32^ unifying to a definition of splenomegaly for spleen length >mean + 2SD in pediatric and ≥13.0 cm in adult patients.

### Statistics

Forty-nine adult patients were identified. Data sets of 45 patients with at least one clinical visit at age 18 to 34 years were included in the cross-sectional analysis. In order to unify the time point of cross-sectional analysis, the visit closest to the age of 26 years was chosen for analysis regarding general patient characteristics as well as kidney and liver phenotype with respect to clinical and sonographic characteristics (“analysed visit”). Four patients had no documented visit at age 18 to 34 years and were only considered in the longitudinal analysis of organ replacement therapies (Fig. 4). Complications occurring in the 45 patients were reported in a time-to-first-event-manner. Continuous variables with normal distribution were expressed as mean (±SD), with non-parametric distribution as median (range or interquartile range (IQR)). Counts and proportions were expressed as number/total number informative cases (percentage). Informative cases were defined as cases with sufficient information for a specific analysis. Numbers therefore vary according to data availability. Survival rates were estimated by the Kaplan-Meier method. The analysis was conducted using R 3.4.2.

## Supplementary information

Supplementary Figure S1

## Data Availability

The data that support the findings of this study are available, on reasonable request, from the corresponding author.

## References

[1] Ward CJ (2002). The gene mutated in autosomal recessive polycystic kidney disease encodes a large, receptor-like protein. Nat. Genet..

[2] Onuchic LF (2002). PKHD1, the polycystic kidney and hepatic disease 1 gene, encodes a novel large protein containing multiple immunoglobulin-like plexin-transcription-factor domains and parallel beta-helix 1 repeats. Am. J. Hum. Genet..

[3] Hildebrandt F, Benzing T, Katsanis N (2011). Ciliopathies. N. Engl. J. Med..

[4] Lu Hao, Galeano Maria C Rondón, Ott Elisabeth, Kaeslin Geraldine, Kausalya P Jaya, Kramer Carina, Ortiz-Brüchle Nadina, Hilger Nadescha, Metzis Vicki, Hiersche Milan, Tay Shang Yew, Tunningley Robert, Vij Shubha, Courtney Andrew D, Whittle Belinda, Wühl Elke, Vester Udo, Hartleben Björn, Neuber Steffen, Frank Valeska, Little Melissa H, Epting Daniel, Papathanasiou Peter, Perkins Andrew C, Wright Graham D, Hunziker Walter, Gee Heon Yung, Otto Edgar A, Zerres Klaus, Hildebrandt Friedhelm, Roy Sudipto, Wicking Carol, Bergmann Carsten (2017). Mutations in DZIP1L, which encodes a ciliary-transition-zone protein, cause autosomal recessive polycystic kidney disease. Nature Genetics.

[5] Harris PC, Torres VE (2009). Polycystic kidney disease. Annu. Rev. Med..

[6] Mekahli D (2016). Kidney Versus Combined Kidney and Liver Transplantation in Young People With Autosomal Recessive Polycystic Kidney Disease: Data From the European Society for Pediatric Nephrology/European Renal Association-European Dialysis and Transplant (ESPN/ERA-EDTA) Registry. Am. J. Kidney Dis. Off. J. Natl. Kidney Found..

[7] Bergmann C (2005). Clinical consequences of PKHD1 mutations in 164 patients with autosomal-recessive polycystic kidney disease (ARPKD). Kidney Int..

[8] Guay-Woodford LM, Desmond RA (2003). Autosomal recessive polycystic kidney disease: the clinical experience in North America. Pediatrics.

[9] Büscher R (2014). Clinical manifestations of autosomal recessive polycystic kidney disease (ARPKD): kidney-related and non-kidney-related phenotypes. Pediatr. Nephrol. Berl. Ger..

[10] Telega G, Cronin D, Avner ED (2013). New approaches to the autosomal recessive polycystic kidney disease patient with dual kidney-liver complications. Pediatr. Transplant..

[11] Adeva M (2006). Clinical and molecular characterization defines a broadened spectrum of autosomal recessive polycystic kidney disease (ARPKD). Medicine (Baltimore).

[12] Ito Y (2017). Renal histology and MRI findings in a 37-year-old Japanese patient with autosomal recessive polycystic kidney disease. Clin. Nephrol..

[13] Fonck C, Chauveau D, Gagnadoux MF, Pirson Y, Grünfeld JP (2001). Autosomal recessive polycystic kidney disease in adulthood. Nephrol. Dial. Transplant. Off. Publ. Eur. Dial. Transpl. Assoc. - Eur. Ren. Assoc..

[14] Banks N (2015). Pregnancy in autosomal recessive polycystic kidney disease. Arch. Gynecol. Obstet..

[15] Gunay-Aygun M (2010). Correlation of kidney function, volume and imaging findings, and PKHD1 mutations in 73 patients with autosomal recessive polycystic kidney disease. Clin. J. Am. Soc. Nephrol. CJASN.

[16] Chalhoub V, Abi-Rafeh L, Hachem K, Ayoub E, Yazbeck P (2013). Intracranial aneurysm and recessive polycystic kidney disease: the third reported case. JAMA Neurol..

[17] Neumann HP, Krumme B, van Velthoven V, Orszagh M, Zerres K (1999). Multiple intracranial aneurysms in a patient with autosomal recessive polycystic kidney disease. Nephrol. Dial. Transplant. Off. Publ. Eur. Dial. Transpl. Assoc. - Eur. Ren. Assoc..

[18] Martínez V (2016). Autosomal recessive polycystic kidney disease diagnosed in a 39 year-old women with kidney failure and cramps. Nefrol. Publicacion Of. Soc. Espanola Nefrol..

[19] Yoshida T, Hiratsuka K, Yamashita M, Matsui A, Hayashi M (2015). Posterior reversible encephalopathy syndrome in a uremic patient with autosomal recessive polycystic kidney disease. CEN Case Rep..

[20] Taneda S (2012). An autopsy case of clinically un-diagnosed autosomal recessive polycystic kidney disease in 77-year-old male. Pathol. Int..

[21] Ebner K (2015). Rationale, design and objectives of ARegPKD, a European ARPKD registry study. BMC Nephrol..

[22] Ebner, K., Schaefer, F., Liebau, M. C. & ARegPKD Consortium. Recent Progress of the ARegPKD Registry Study on Autosomal Recessive Polycystic Kidney Disease. *Front. Pediatr*. **5**, 18 (2017). 10.3389/fped.2017.00018 PMC5327862 28296980

[23] Richards S (2015). Standards and guidelines for the interpretation of sequence variants: a joint consensus recommendation of the American College of Medical Genetics and Genomics and the Association for Molecular Pathology. Genet. Med. Off. J. Am. Coll. Med. Genet..

[24] Pottel H (2016). An estimated glomerular filtration rate equation for the full age spectrum. Nephrol. Dial. Transplant. Off. Publ. Eur. Dial. Transpl. Assoc. - Eur. Ren. Assoc..

[25] Scholbach T, Weitzel D (2012). Body-surface-area related renal volume: a common normal range from birth to adulthood. Scientifica.

[26] Weitzel D. (1997). Nieren und ableitende Harnwege. Die Ultraschalluntersuchung des Kindes.

[27] Spiegl G, Jeanty P, Kittel F, Struyven J (1982). Ultrasonic measure of the normal kidney. J. Belge Radiol..

[28] Ultraschalldiagnostik in Pädiatrie und Kinderchirurgie - Thieme.de - Thieme Webshop - Deeg, Karl-Heinz, Hofmann, Volker & Hoyer, Peter Friedrich. *Thieme Webshop* Available at: http://www.thieme.de/shop/Paediatrie/Deeg-Hofmann-Hoyer-Ultraschalldiagnostik-in-Paediatrie-und-Kinderchirurgie-9783132424661/p/000000000178740105 (Accessed: 14th June 2018).

[29] Kratzer W (2003). Factors affecting liver size: a sonographic survey of 2080 subjects. J. Ultrasound Med. Off. J. Am. Inst. Ultrasound Med..

[30] Rosenberg HK (1991). Normal splenic size in infants and children: sonographic measurements. AJR Am. J. Roentgenol..

[31] El Sharkawy E (1997). Ultra sonographic measurements of the normal liver and spleen among Egyptians 10–50 years old. J. Egypt. Public Health Assoc..

[32] Yazdanpanah Y (1997). Organometric investigations of the spleen and liver by ultrasound in Schistosoma mansoni endemic and nonendemic villages in Senegal. Am. J. Trop. Med. Hyg..

