# Supplementary material for: Clinical courses and complications of young adults with Autosomal Recessive Polycystic Kidney Disease (ARPKD)
Source: Sci Rep. 2019 May 28;9:7919. doi: 10.1038/s41598-019-43488-w (PMC6538621; doi:10.1038/s41598-019-43488-w)
Supplement: Supplementary file 1 — Supplementary Figure S1 [file 41598_2019_43488_MOESM1_ESM.pdf]

Clinical courses and complications of young adults with Autosomal Recessive Polycystic Kidney Disease (ARPKD)

Kathrin Burgmaier[1], Samuel Kilian[2], Bert Bammens[3,4], Thomas Benzing[5,6], Heiko Billing[7], Anja Büscher[8], Matthias Galiano[9], Franziska Grundmann[5], Günter Klaus[10], Djalila Mekahli[11,12], Laurence Michel-Calemard[13], Gordana Milosevski-Lomic[14], Bruno Ranchin[15], Katja Sauerstein[9], Susanne Schaefer[16], Rukshana Shroff[17], Rosalie Sterenborg[17], Sarah Verbeeck[12], Lutz T. Weber[1], Dorota Wicher[18], Elke Wühl[16], Jörg Dötsch[1], Franz Schaefer[16], Max C. Liebau[1,6]*

[1] Department of Pediatrics, University Hospital of Cologne, Cologne, Germany

[2] Institute of Medical Biometry and Informatics, University of Heidelberg, Heidelberg, Germany

[3] Department of Microbiology and Immunology, Laboratory of Nephrology, KU Leuven, Leuven, Belgium

[4] Department of Nephrology, Dialysis and Renal Transplantation, University Hospitals Leuven, Leuven, Belgium

[5] Department II of Internal Medicine, University Hospital of Cologne, Cologne, Germany

[6] Center for Molecular Medicine, University Hospital of Cologne, Cologne, Germany

[7] Children's University Hospital Tuebingen, Department of General Pediatrics and Hematology/Oncology, Tuebingen, Germany

[8] Department of Pediatrics II, University Hospital Essen, Essen, Germany

[9] Department of Pediatrics and Adolescent Medicine, Hospital of the Friedrich-Alexander-University Erlangen-Nürnberg (FAU), Erlangen, Germany

[10] KfH Center of Paediatric Nephrology, University Hospital of Marburg, Marburg, Germany

[11] PKD Research Group, Department of Development and Regeneration, KU Leuven, Leuven, Belgium

[12] Department of Pediatric Nephrology, University Hospitals Leuven, Leuven, Belgium

[13] Service Biochimie Biologie Moléculaire Grand Est, Hospices Civils de Lyon, Bron Cedex, France

[14] Department of Nephrology, University Children's Hospital, Belgrade, Serbia

[15] Pediatric Nephrology Unit, Hôpital Femme Mere Enfant, Hospices Civils de Lyon, Lyon, France

[16] Division of Pediatric Nephrology, Center for Pediatrics and Adolescent Medicine, University of Heidelberg, Heidelberg, Germany

[17] Great Ormond Street Hospital for Children NHS Foundation Trust, London, United Kingdom

[18] Department of Medical Genetics, The Children's Memorial Health Institute, Warsaw, Poland

*max.liebau@uk-koeln.de

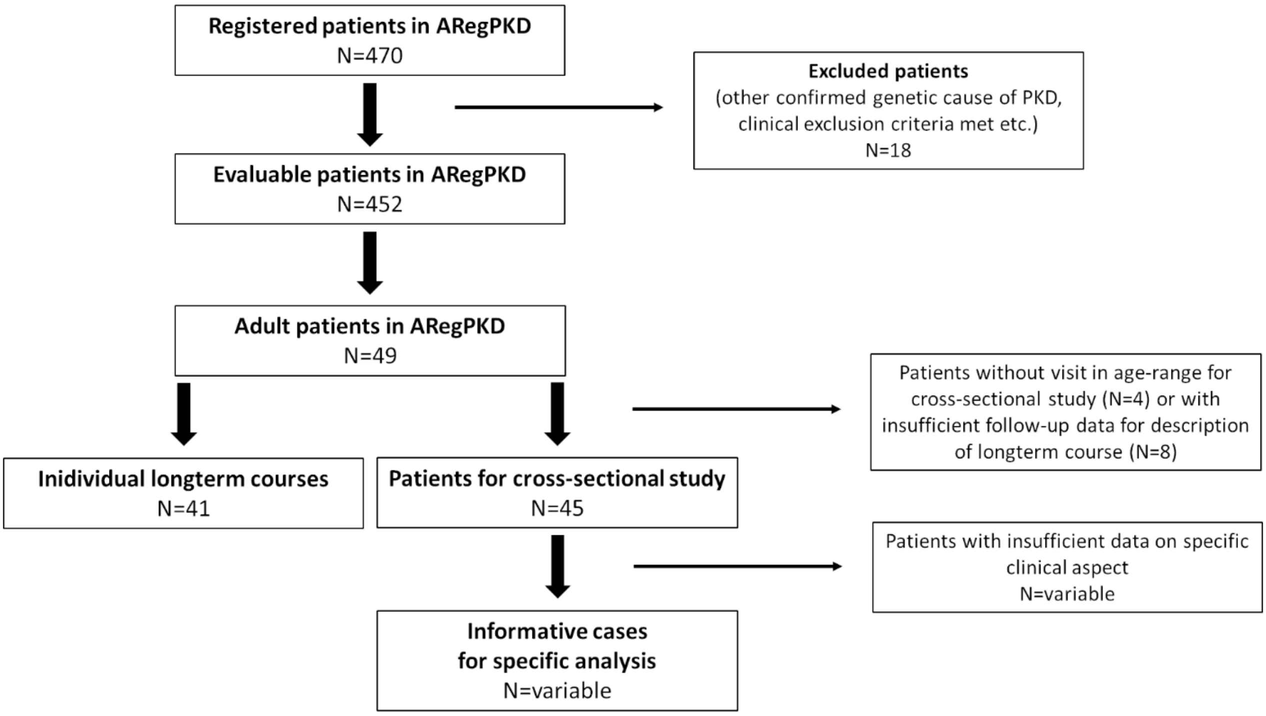

Supplementary Figure S1. Flow chart of the patient selection process.
